# Recovery of a migrated stent during endoscopic ultrasound-guided pancreatic pseudocyst drainage using a lithotripsy basket

**DOI:** 10.1055/a-2781-6016

**Published:** 2026-02-24

**Authors:** Kiyoyuki Kobayashi, Hideki Kamada, Manabu Yamada, Daisuke Namima, Naoki Fujita, Hiroki Yamana, Hideki Kobara

**Affiliations:** 1 38078 Division of Innovative Medicine for Hepatobiliary and Pancreatology, Faculty of Medicine, Kagawa University, Kagawa, Japan; 2 38078 Department of Gastroenterology and Neurology, Faculty of Medicine, Kagawa University, Kagawa, Japan

**Fig. 1:**
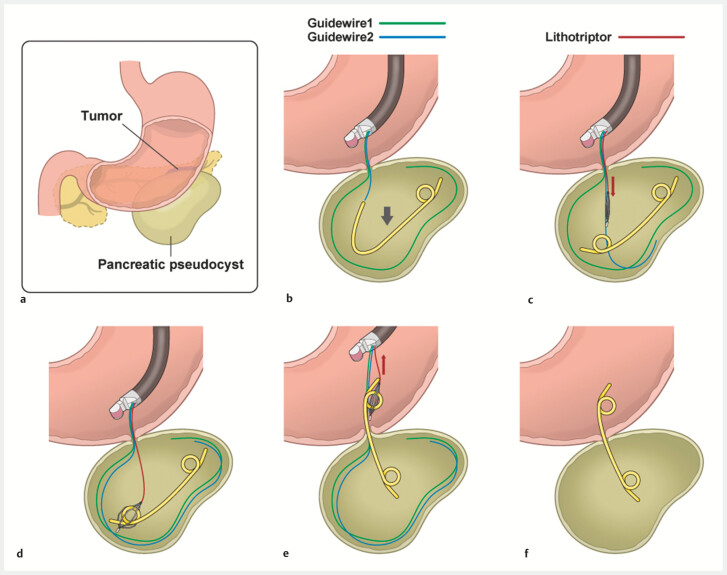
Step-by-step schematic of the lithotripsy basket rescue approach for addressing intracystic plastic stent migration during endoscopic ultrasound-guided pancreatic pseudocyst drainage. **a** The anatomical relationship between our patient’s pancreatic tail cancer and pancreatic pseudocyst. **b** Inadvertent complete migration of the double-pigtail stent into the pseudocyst, with two guidewires (green and blue) remaining across the gastric wall. **c** Insertion of a monorail-type lithotripsy basket (red) along the guidewire into the cyst. **d** Removal of the guidewires within the cyst to ensure optimal basket manipulation. **e** Capture of the proximal stent end with the four-wire basket and withdrawal into the gastric lumen. **f** Successful repositioning of the stent into the intended trans-gastric drainage position. Source: Medical Education Inc.

Endoscopic ultrasound-guided pancreatic pseudocyst drainage (EUS-PPD) is an established therapeutic approach, and the use of lumen-apposing metal stents (LAMSs) has increased in recent years. However, EUS-PPD with plastic stents (PSs) remains a valuable and cost-effective option for fluid-predominated pseudocysts. Recent studies have demonstrated comparable outcomes between the LAMS and the PS for pancreatic pseudocysts, with no significant differences in adverse events 1 2 . However, stent migration remains a common complication that requires appropriate management 3 4 . Herein, we present an effective salvage technique using a readily available lithotripsy basket ( Fig. 1 ).

**Fig. 2:**
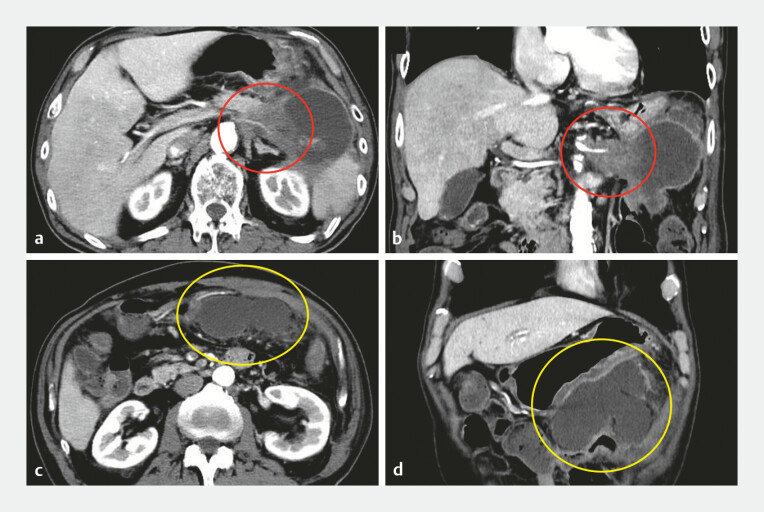
Pre-procedural contrast-enhanced computed tomography. **a** An axial image showing the unresectable pancreatic tail cancer (red circle) and associated pancreatic pseudocyst. **b** Coronal reconstruction showing the spatial relationship between the tumor and the pseudocyst (red circle). **c** An axial image at a lower level highlighting the large fluid-predominant pseudocyst (yellow circle). **d** A coronal view of the pseudocyst (yellow circle), ~10 cm in diameter, located adjacent to the stomach.

A 70-year-old man with unresectable pancreatic tail cancer was admitted for abdominal pain caused by an infected pancreatic pseudocyst. EUS-PPD was performed for the ~10 cm, fluid-predominant collection ( Fig. 2 ). Following puncture with a 19 G needle, 0.025” and 0.035” guidewires were inserted and the puncture site was balloon-dilated. During the insertion of a 7 Fr, 7 cm double-pigtail PS, the entire stent inadvertently migrated into the cystic cavity.

**Fig. 3:**
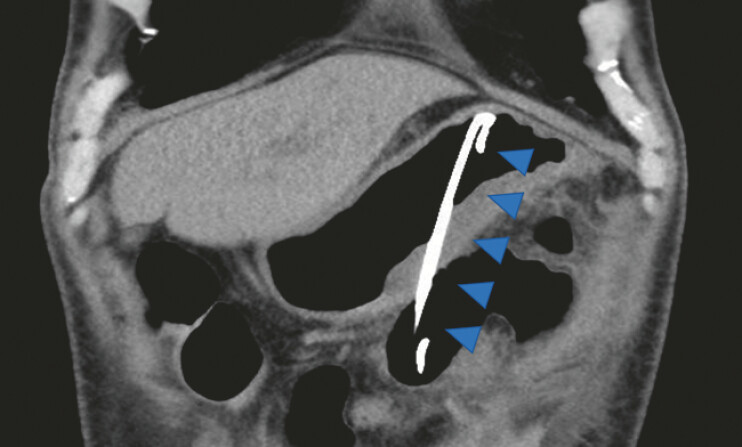
A coronal computed tomography image after the rescue procedure, demonstrating the double-pigtail plastic stent (blue arrowheads) located in the intended trans-gastric position to provide adequate drainage of the pancreatic pseudocyst.

To retrieve it, a monorail-type guidewire-guided LithoCrush V mechanical lithotripsy basket (Olympus Medical Systems, Tokyo, Japan) was advanced into the target site within the cyst. After removing the guidewire, the basket was deployed to capture the proximal stent end, and the stent was withdrawn into the stomach. The basket was detached, leaving the stent in the intended position for gastric-pseudocyst drainage ( Fig. 3 ; Video 1 ). Symptom improvement was achieved soon afterward.

Complete recovery of a migrated stent during endoscopic ultrasound-guided pancreatic pseudocyst drainage using a lithotripsy basket.Video 1

This technique offers three advantages. First, guidewire guidance facilitates easy navigation to the target location; second, the monorail system enables intracystic guidewire removal to ensure optimal basket maneuverability; third, the four-wire basket facilitates secure grasping and controlled stent release. This technique therefore facilitates stent repositioning to the originally intended location even if inadvertent migration occurs.

This rescue technique provides a simple, clinically effective, and cost-efficient method for managing intracystic PS migration during EUS-PPD.

Endoscopy_UCTN_Code_CPL_1AL_2AD
